# Interoperability in universal healthcare systems: insights from Brazil's experience integrating primary and hospital health care data

**DOI:** 10.3389/fdgth.2025.1622302

**Published:** 2025-08-18

**Authors:** Michelle Fernandez, Heider Aurélio Pinto, Luisa M. M. Fernandes, João André Santos de Oliveira, Ana Maria Freire de Souza Lima, José Santos Souza Santana, Arthur Chioro

**Affiliations:** ^1^ Institute of Political Science, University of Brasília, Brasília, Brazil; ^2^ Department of Preventive and Social Medicine, Federal University of Bahia, Salvador, Brazil; ^3^ Research Center Renê Rachou/Fiocruz, Belo Horizonte, Brazil; ^4^ Department of Family Health and Occupational Therapy, Federal University of Bahia, Salvador, Brazil; ^5^ Department of Social and Pediatric Dentistry, Federal University of Bahia, Salvador, Brazil; ^6^ Department of Preventive Medicine, Federal University of São Paulo, São Paulo, Brazil

**Keywords:** digital health, interoperability, electronic health record, health information systems, Brazilian Unified Health System

As 2030 approaches, the global community faces increasing pressure to achieve the Sustainable Development Goals (SDGs). A critical element in this endeavor is the digital transformation of healthcare systems, which is essential to addressing challenges such as the epidemiological transition and rising healthcare costs (1, 2). The 21st century has brought significant advancements in Information and Communication Technologies (ICT), including Artificial Intelligence (AI), reshaping healthcare delivery, surveillance, and management (3).

The Brazilian healthcare system (Sistema Único de Saúde—SUS), established in 1988 on the principles of universality, equity, comprehensiveness, and regionalization, faces historical challenges related to the fragmentation of clinical data. While digitalization has expanded the accessibility of information, the coexistence of various electronic health record systems—governmental and private-owned—that lack interoperability hinders the ability of healthcare professionals and managers to make cost-effective decisions that lead to timely improvements in patient care (3–5). Interoperability is essential for overcoming these challenges, considering it contributes to applying analytical tools, such as predictive analytics and AI applications (5–8).

Notable progress has been made through collaborative initiatives that integrate digital health infrastructure and promote systemic innovation. A leading example is the partnership between the city of Recife, with over 1.5 million residents, and the Brazilian Company of Hospital Services (Ebserh), which manages over 45 federal university hospitals. Together, they established Brazil's first data federation between two interoperability platforms—one municipal and the other federal—connecting data from primary, secondary, and tertiary levels of care. This integration enables the secure exchange of information on vaccinations, diagnostic tests, medications, scheduling, and clinical records, facilitating continuity of care throughout the patient journey.

In Recife, healthcare professionals across any of the services within the network have access to citizens' clinical data through a data repository that interconnects information from four governmental and private clinical electronic health records and eight national information systems. The municipal data is sourced from 202 local primary healthcare services covering over 60% of the population. This integration was made possible through adopting the Electronic Health Platform (iPES), locally rebranded as *Saúde Conectada (Connected Health)* and implemented by its state-owned enterprise, the Municipal Informatics Company (Emprel). The iPES resulted from a request for proposal (RFP) under the 2016 innovation law by a state company, aiming to develop a solution to ensure interoperability among various health applications and systemsent (5, 9). The main challenge was the need of implementing technological infrastructure within all health care units before health professionals could start using iPES.

Simultaneously, Ebserh implemented a platform with similar technology and architecture, enabling clinical data interoperability from its hospitals covering approximately 25 million patients across the country. Through the data federation between these platforms, citizens and healthcare professionals can access clinical records generated in any of Recife's 202 primary healthcare services and Ebserh-managed hospitals. This integration can enhance diagnostic accuracy and timeliness, improve continuity of care, prevent unnecessary procedures, and optimize resource allocation (3, 7, 8).

Both platforms utilize a cloud-based, service-oriented architecture with interoperability buses, API (Application Programming Interface) management, access control, and identity management. They incorporate services like Master Patient Index, Clinical Document Repository, consent management, terminology management, and metadata governance. Adhering to international interoperability standards such as HL7-FHIR and OpenEHR, allows for adaptability to various clinical and administrative use cases. The platforms already had the same semantics, however, differences in the policies for accessing user data by healthcare professionals (more restrictive at Ebserh) required adjustments and caused a small delay in data federation. Data storage is managed in FHIR-native data lakes, equipped with APIs that facilitate connections for sending and receiving normalized data—either identified or anonymized—while ensuring compliance with personal data protection regulations.

This experience underscores the importance of collaboration among institutions in advancing digital health, however such collaboration is only possible when there is political will. It demonstrates that system integration is feasible, even within Brazil's complex federative healthcare context, where health policy responsibilities are shared between federal and subnational governments (9). The success of system integration in such intricate healthcare systems should inspire confidence in digital health's future, illustrating that, with the right strategies and collaboration, digital health can thrive in even the most challenging environments. The same model of data federation agreements could be replicated as long as health care units and/or hospitals use electronic health records and the governor entities are willing to share data focused on patient care within the data sharing law in Brazil.

The observed results extend beyond clinical improvements and underscore the transformative potential of digital health in Brazil. The integration of interoperable platforms has enhanced care coordination, facilitated access to longitudinal patient data, and supported better clinical decision-making. Moreover, by enabling the safe exchange of structured health information, these initiatives have improved the efficiency of service delivery and laid the foundation for more robust population health management strategies (10). From a governance perspective, this experience demonstrates that political commitment, aligned with technical and institutional capacity, is important to scaling digital innovations in complex federative systems.

In this way, the Brazilian experience contributes to the growing repertoire of strategies adopted by countries across Europe, Asia, and the Americas to advance the achievement of the SDGs (11–15), particularly by enhancing national governance of digital health and strengthening public-private partnerships to address challenges related to technological development and financing.

Interoperability has played a crucial role in overcoming data fragmentation, with significant potential to enhance care continuity, enable large-scale data analysis, support strategic decision-making, and optimize case and resource management. Moreover, this experience demonstrates that effective planning and innovation allow cost-effective implementation of digital health policies that contribute to the sustainable development of the healthcare system.

In conclusion, Brazil's digital health initiatives underscore the strategic importance of interoperability in strengthening health systems and enhancing the quality and efficiency of patient care in complex institutional settings. By promoting inter-institutional collaboration and fostering innovation, these efforts contribute to advancing broader health system goals and improving service delivery at the national level.
